# Ground Reaction Forces of Dressage Horses Performing the Piaffe

**DOI:** 10.3390/ani11020436

**Published:** 2021-02-08

**Authors:** Hilary Mary Clayton, Sarah Jane Hobbs

**Affiliations:** 1 Large Animal Clinical Sciences, Michigan State University, East Lansing, MI 48824, USA; 2 School of Sport and Health Sciences, University of Central Lancashire, Preston PR1 2HE, UK; sjhobbs1@uclan.ac.uk

**Keywords:** ground reaction force, force vector diagram, Grand Prix dressage, balance

## Abstract

**Simple Summary:**

One of the most difficult movements performed by dressage horses is the piaffe, in which the horse raises and lowers alternating diagonal limb pairs while remaining in place. Piaffe is an artificial movement that requires good balance. Knowledge of the unique stresses on the horse’s limbs and body during the performance of the piaffe are needed to understand the mechanics of the movement and the implications for injury. In this study, we used force plates to measure ground reaction forces (GRFs) in the vertical, longitudinal and transverse directions in seven highly trained horses performing the piaffe. The results showed that the hindlimbs carried relatively more weight in the piaffe than in trot or passage, though the peak vertical GRF was significantly higher in the forelimbs. The forces acting in the horizontal plane showed considerable variability from step-to-step within individual horses. This was thought to represent the difficulty of maintaining balance when the horse stands on one diagonal pair of limbs.

**Abstract:**

The piaffe is an artificial, diagonally coordinated movement performed in the highest levels of dressage competition. The ground reaction forces (GRFs) of horses performing the piaffe do not appear to have been reported. Therefore, the objective of this study was to describe three-dimensional GRFs in ridden dressage horses performing the piaffe. In-ground force plates were used to capture fore and hindlimb GRF data from seven well-trained dressage horses. Peak vertical GRF was significantly higher in forelimbs than in the hindlimbs (7.39 ± 0.99 N/kg vs. 6.41 ± 0.64 N/kg; *p* < 0.001) with vertical impulse showing a trend toward higher forelimb values. Peak longitudinal forces were small with no difference in the magnitude of braking or propulsive forces between fore and hindlimbs. Peak transverse forces were similar in magnitude to longitudinal forces and were mostly directed medially in the hindlimbs. Both the intra- and inter-individual variability of longitudinal and transverse GRFs were high (coefficient of variation 25–68%). Compared with the other diagonal gaits of dressage horses, the vertical GRF somewhat shifted toward the hindlimbs. The high step-to-step variability of the horizontal GRF components is thought to reflect the challenge of balancing on one diagonal pair of limbs with no forward momentum.

## 1. Introduction

Piaffe is a highly collected, cadenced, elevated diagonal movement that gives the impression of remaining in place [1]. It is recognized as one of the most difficult movements in the sport of dressage and failure to learn how to perform the piaffe is one of the reasons that horses fail to attain the highest level of competition.

The diagonally coordinated gaits of dressage horses are the trot, passage and piaffe. The trot is a forward-moving gait with four distinct variations performed at a range of speeds (mean ± SD) from extended trot (4.93 ± 0.14 m/s), through medium trot (4.47 ± 0.23 m/s), working trot (3.61 ± 0.10 m/s), and collected trot (3.20 ± 0.28 m/s) [2]. Passage is a slow majestic type of trot performed at a slower speed in the range of 1.2–1.9 m/s [3,4,5] and characterized by a hovering motion of the limbs at their maximal height during the swing phase. As locomotor speed decreases, the maintenance of balance becomes more challenging due to a greater reliance on static equilibrium. Piaffe has little, if any, forward velocity. In the Intermediare II dressage test, it is permissible for the horse to progress forwards up to 10 cm per step of piaffe, equivalent to a speed of ~0.2 m/s, whereas in the Grand Prix test, the steps should be performed in place [1]. One of the reasons horses struggle to learn the piaffe is that, as translational motion decreases, the reliance on static equilibrium increases and it becomes progressively more difficult to raise a diagonal pair of limbs without becoming unbalanced. As a general rule, a decrease in speed is accompanied by longer stance durations and longer periods of overlap between the limbs as has been previously reported for the diagonally coordinated equine gaits [3,4,6]. Inter-limb overlaps enlarge the base of support, facilitating a greater reliance on static equilibrium. Furthermore, the ground reaction forces (GRFs) may be adjusted both in magnitude and direction throughout the diagonal stance to control the rotational moments of the horse’s body around the center of mass (COM) [5]. Previous studies of diagonally synchronized gaits have shown that strategies for maintaining balance include adjusting footfall timings, adjusting hoof placements and changing the GRF distribution between the fore- and hindlimbs [5,7].

As a first step toward characterizing the balancing mechanisms in the piaffe, this study measured the GRFs in seven elite dressage horses performing the piaffe. The extraction of force data was complicated by the absence of aerial phases [3,4,6], which resulted in temporal overlaps between the stance phases of contralateral limbs giving rise to periods of tripedal and even quadrupedal support. Furthermore, since the hooves are raised and lowered with minimal forward progression in each step, the spatial separation of contralateral hooves is small and consequently, it is unusual to have only a single hoof on a force plate for its entire stance duration. The extraction of GRF data for a single limb is one of the challenges addressed in this study.

The objective was to describe three-dimensional GRF data in dressage horses performing the piaffe. We anticipated that, compared with the values reported for passage [5], peak vertical forces and vertical impulses will be lower in all limbs since the piaffe has no suspension phases; longitudinal forces are expected to be low in association with the lack of forward progression; and medially directed GRFs are expected to increase as a means of enhancing balance.

## 2. Materials and Methods

Data for this study were produced at Utrecht University and Michigan State University between 1999 and 2013. The protocol was approved by the Michigan State University institutional animal care and use committee (approval number 02/08-020-00).

The subjects were seven sound, highly trained dressage horses, three Dutch warmbloods (W1, W2, W3) and four Lusitanos (L1, L2, L3, L4). The body mass of the Dutch warmbloods was (mean ± SD) 715 ± 33 kg with W1 and W3 being ridden by the same female rider with a body mass of 70 kg and W2 ridden by a male rider with mass 85 kg. All had competed at the Grand Prix level and W1 was a multiple Olympic medalist. The Lusitanos had body mass (556 ± 21 kg) and were all ridden by the same female rider with a mass of 61.5 kg. HorsesHorses L1 and L2 were successful competitors at the Grand Prix level, L3 and L4 were trained in academic dressage L3 to Prix St Georges and L4 to Grand Prix level.

The horses were warmed up in a dressage arena according to the rider’s usual routine. Data were then collected as each horse performed steps of the piaffe on one or more force plates embedded within a rubberized runway and covered with the same material as the runway. Horses approached the force plates in passage and made a transition to the piaffe prior to the forelimbs stepping onto the first force plate. Data for the Dutch warmbloods were collected using a single force plate (Type Z4852C, Kistler Corp., Winterthur, Switzerland) recording at 100 Hz (W1) or 200 Hz (W2, W3). Data for the Lusitanos were collected using four force plates in series (two FP60120 and two FP6090, Bertec Corporation, Columbus, OH, USA) recording at 1000 Hz (L1) or 960 Hz (L2, L3, L4).

Three retro-reflective markers secured to the wall of each hoof were tracked to assist in identifying hoof contacts and lift offs, and to confirm that the solar surface of the hoof was entirely on the force plate. For the Dutch warmbloods, two video cameras (Panasonic AG450, Matsushita Electric Corp. of America, Secaucus, NJ, USA) recording at 60 Hz were used and for the Lusitanos a 10-camera motion capture system (Motion Analysis Corp., Santa Rosa, CA, USA) recording at 120 Hz and incorporating a video recording was used.

Each trial was evaluated by the rider immediately after it was collected and at a later date the videos were scrutinized by an experienced observer (HMC) who trains at the Grand Prix level and is a former judge. If the quality of the performance was deemed inadequate, the trials were discarded. The remaining trials were screened to check that the horse performed steps of the piaffe with little or no forward progression, with the body aligned along the long axis of the force plate, and with one or more grounded hooves completely on the force plate.

**Figure 1:**
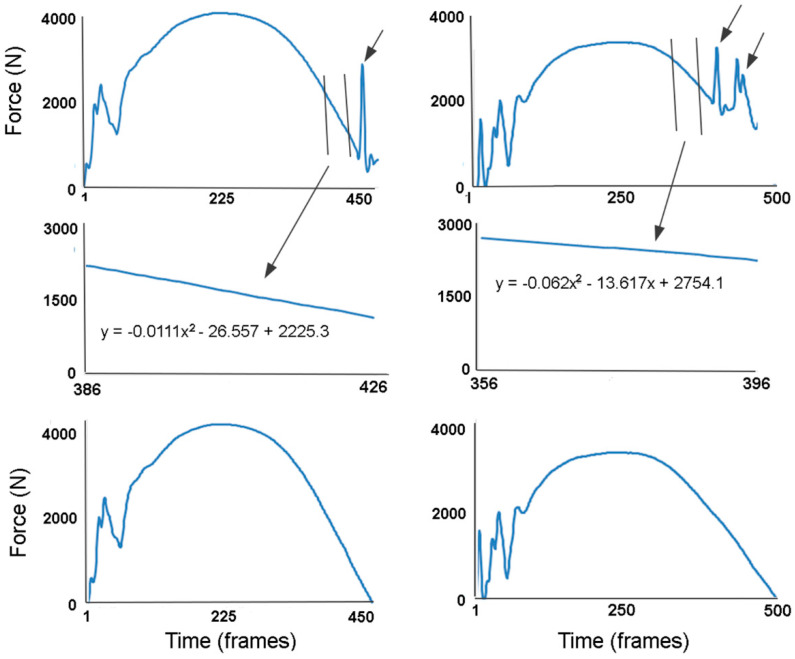
The estimation of vertical ground reaction force during the periods of stance overlap for two stance phases: (**A**) on the left; (**B**) on the right. The upper graph shows the vertical ground reaction force-time graph with impact peaks (indicated with short arrows) in terminal stance, which is associated with the contact of a second hoof on the same force plate. The vertical lines show the part of the trace used to develop a second order polynomial equation shown in the middle graph. The polynomial was used to estimate the terminal part of the vertical ground reaction force trace. The lower graph shows the estimated vertical force-time profiles. Each polynomial was unique and was determined using an iterative process in which the selection window of frames was adjusted until a best fit unloading slope with a threshold smaller than ± 100 N at lift off was found.

The observation of video or motion capture data was used to screen trials and remove trials in which none of the hooves had full contact on a force plate. Trials in which a single hoof was on the force plate during its entire stance phase without other hooves being in full or partial contact with the same plate were selected first for analysis. These were relatively few in number because in piaffe the contralateral hooves were placed in close spatial proximity and in the absence of forward motion, are often present on the same force plate simultaneously. Trials in which one hoof was in full contact with a force plate and a second hoof then contacted the same force plate were identified based on the presence of impact peaks in the vertical GRF (GRFv) curves of the stance phase limb (Figure 1). In these cases, the hoof lift off was determined from a combination of visual evaluation and a sudden large excursion of the center of pressure (COP) on the force plate.

GRFs for each stance phase were extracted as text files and compiled in Excel. Force-time graphs were produced for the vertical, longitudinal and transverse force components in each stance phase of the fore- and hindlimbs. When contralateral hoof contacts had a short period of overlap (n = 13 stance phases), the vertical GRF curve for the limb in terminal stance was estimated from the first impact spike associated with the contact of the second hoof until lift off, using a unique (to each stance phase) second order polynomial (Figure 1). This was not possible for longitudinal or transverse GRFs, as the traces were too variable to be able to predict the path of the GRF curve.

Vertical GRFs were positive upwards, in the longitudinal direction braking GRFs were negative and propulsive GRFs were positive, and in the transverse direction, the lateral forces were negative and medial forces were positive. For GRFv, the peak value was extracted together with its time of occurrence as a percentage of stance duration. Vertical impulse was calculated by integration of the GRFv curve. For longitudinal and transverse GRF curves, the peak values not associated with impact spikes were extracted, provided they were above a threshold of 0.1 N/kg because in some stance phases, the GRFs fluctuated close to zero and it was not possible to clearly identify peaks within the force traces. When distinct peaks were present, their magnitude and time of occurrence relative to the stance phase were determined. For each stance phase, this provided either one peak in only one direction or two peaks, one in each direction. Since the piaffe is classified as a symmetrical gait, data for the left and right contralateral limbs were pooled as in previous studies of passage [4,5,8]. For each horse, the GRFs were analyzed for four forelimb contacts and four hindlimb contacts, except horse L4 which had four forelimb contacts only. Force data were normalized to the combined mass of the horse and rider.

Force vector diagrams were constructed in the sagittal and frontal planes in Excel (Microsoft Limited, Reading, Berks, UK) with the GRF data normalized to 101 points. Each diagram represented a single stance phase with the component vectors originating at the center of pressure on the force plate and scaled to match the magnitude and direction of the vectors [9]. For each diagram, vector magnitude (VecMag) was calculated by the vector summation of the individual vectors divided by the number of contributing samples. The angle of the summary vector (VecAng) was determined trigonometrically from the components of the vector magnitude and expressed relative to the vertical with positive values directed cranially (sagittal plane) or medially (frontal plane). Summary vectors were plotted to provide a visual interpretation of the mean forces and the values of VecMag and VecAng were calculated. Due to the fact that the horizontal forces include data from the impact spikes of overlapping limbs, they were not statistically evaluated.

Descriptive statistics (mean, SD) were calculated for each variable. If there was a single peak in a longitudinal or transverse GRF curve, the datum for a second peak in the opposite direction was identified as missing. The number of peak values extracted from the dataset are reported for each variable. Statistical comparisons were made between the limbs (fore vs. hind) and between horses using ANOVA, Mann–Whitney U test (fore vs. hind) or Kruskall–Wallis test (between horses) depending on the normality of the data distribution. When significant differences were present between the horses, post hoc Bonferroni adjusted pairwise comparisons were performed. All statistical analyses were performed in SPSS. Significance was set at *p* < 0.05.

## 3. Results

**Figure 2:**
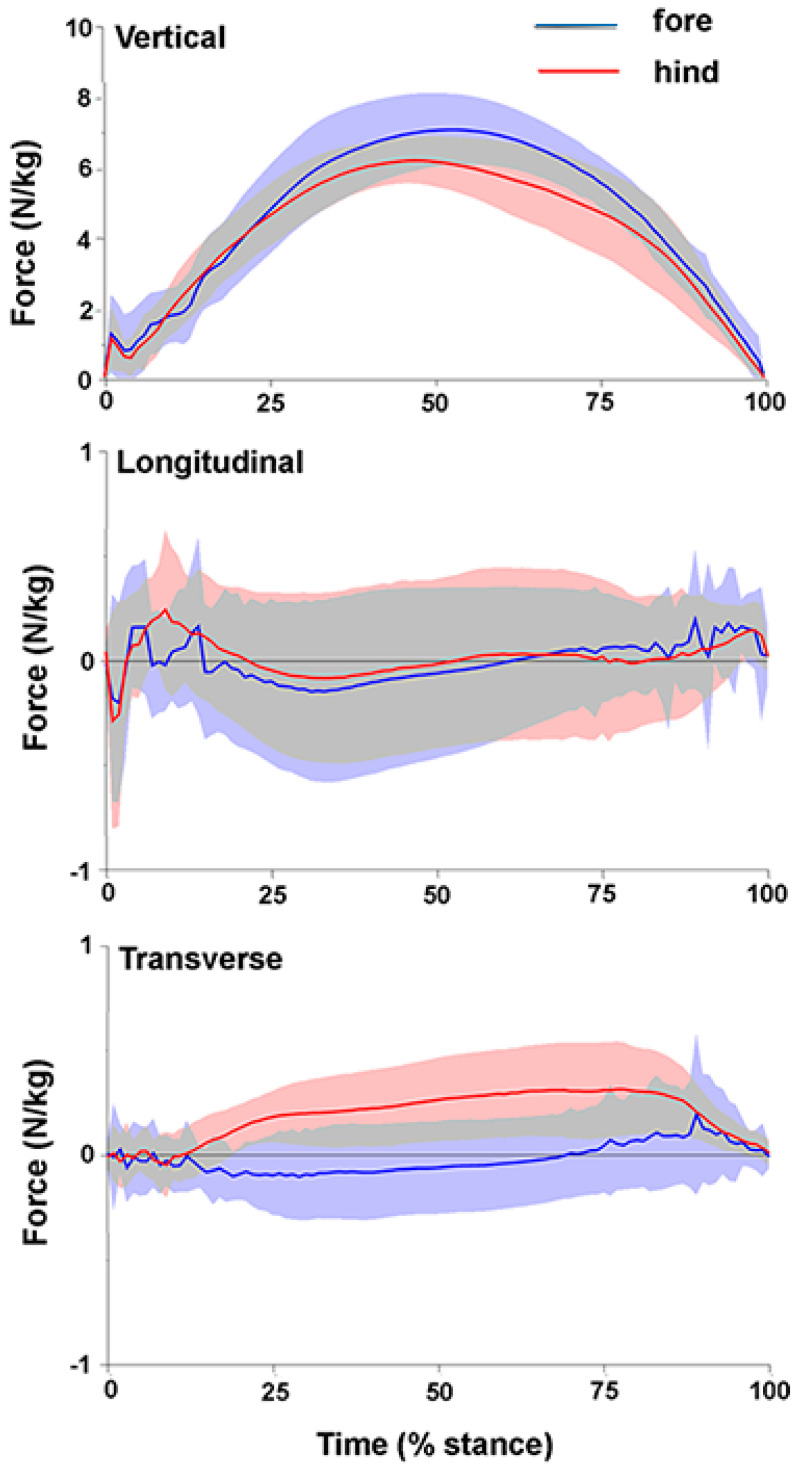
Mean values (bold lines) and SD bands (shaded areas) (N = 7 horses) for vertical (above), longitudinal (middle) and transverse (below) ground reaction forces for the forelimbs (blue) and hindlimbs (red). Overlapping areas of the SD bands are shown in grey.

Mean values ± SD and Coefficient of Variation (CoV) for stance durations, mass-normalized peak values of vertical, longitudinal and transverse GRFs and their times of occurrence, together with vertical impulses are shown in Table 1. Absolute values and standard deviation bands for the vertical, longitudinal and transverse GRF components of the fore- and hind-limbs averaged across all horses are shown in Figure 2.

**Figure 3:**
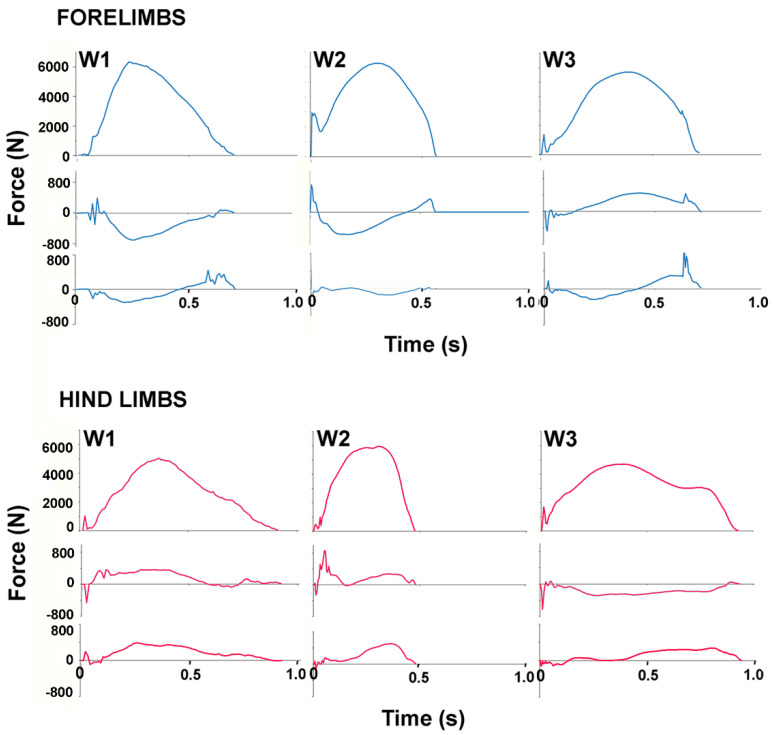
Ground reaction force traces for one example of a stance phase of each warmblood horse: left to right: W1, W2, W3. Vertical (above), longitudinal (middle) and transverse (below) forces for forelimbs (upper panel) and hindlimbs (lower panel).

**Figure 4:**
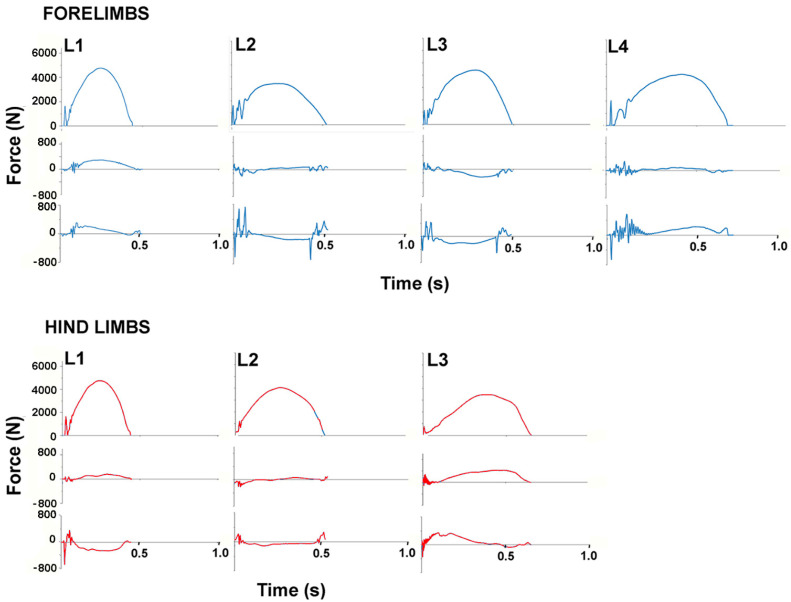
Ground reaction force traces for one typical stance phase of each Lusitano horse: left to right: L1, L2, L3, L4. Vertical (above), longitudinal (middle) and transverse (below) forces for forelimbs (upper panel) and hindlimbs (lower panel).

Stance durations did not differ between the fore- and hindlimbs but there were significant differences between horses. The stance durations of W1 and W3 are clearly longer than in the other horses in Figure 3 and Figure 4_._, especially for the hindlimbs. The only variable that differed significantly between the fore- and hindlimbs (Table 1) was normalized peak GRFv which was higher in the forelimbs (*p* < 0.001). Vertical impulse showed a trend (*p* = 0.084) toward being higher in the forelimbs. The traces for GRFv in Figure 2 show similar loading rates in the fore- and hindlimbs in the first 25% of stance after which the forelimb curve rises above that of the hindlimb. Even in the mass normalized data in Figure 2, the SD bands are wide, especially for the longitudinal forces.

Including the weights of the riders, all Warmbloods were heavier than all Lusitanos, and this should be borne in mind when looking at the absolute GRFs in Figure 3 and Figure 4. The mean stance duration for all horses did not differ between fore- and hindlimbs but individual horses sometimes showed quite marked differences (see horses 1 and 3 in Figure 3). The GRFv graphs show impact spiking followed by a smooth rise to a single peak during the middle part of stance in all horses, though there are obvious differences in the shape of the individual curves. Significant inter-horse differences (*p* < 0.05) were found for mass normalized mean peak GRFv, time of peak GRFv and vertical impulse.

**Figure 5:**
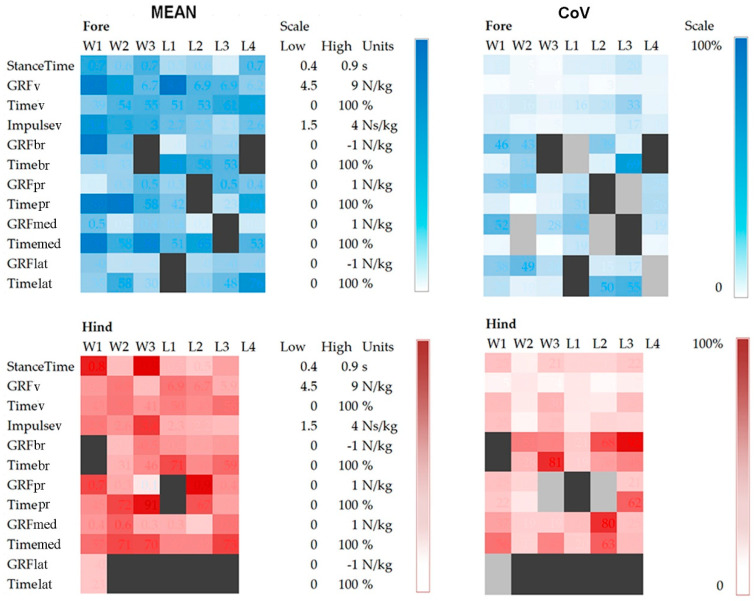
Heat maps showing inter-horse variability represented by the mean values (left) and intra-horse variability represented by coefficients of variation (CoV) (right). Stance time and GRF variables are on the vertical axis, individual horses are on the horizontal axis. Color-coding is on a sliding scale with darker shades indicating greater magnitude. Dark grey cells have no data and light grey cells in CoV maps have only one datum point so the CoV could not be calculated.

In the longitudinal direction, the mass normalized peak values of the braking component (GRFbr) and the propulsive component (GRFpr) were low and had large CoV; neither the values nor their times of occurrence differed between fore- and hindlimbs (Table 1). The mean longitudinal GRF trace showed initial impact spiking, especially in the forelimbs, followed by a braking (negative) phase and then a propulsive (positive) phase. The SD bands are wide. Inter-horse variability is reflected in the varied shapes of the longitudinal GRF curves illustrated in Figure 4 and Figure 5. Significant differences (*p* < 0.05) were present between the horses for the mass normalized mean peak GRFpr and the times of occurrence of the braking and propulsive longitudinal force peaks (Table 1).

In the transverse direction, the mean mass normalized GRF traces were slightly medially directed through most of stance in the hindlimbs and slightly laterally directed during the early and midstance in the forelimbs (Figure 2). Figure 4 and Figure 5 show a range of transverse GRF patterns in the fore- and hindlimbs that resulted in a high CoV (Table 1). Peak value for the medial GRF (GRFmed) was significantly different between horses (*p* = 0.015) but only one hindlimb had a measurable peak in the lateral GRF (GRFlat) so a comparison for that variable was not possible.

Significant inter-horse comparisons are shown in Table 2. A greater number of letters depicts a greater number of variables that are significantly different between a pair of horses. For example, W3 vs. L2, and W1 vs. L4 have the greatest number of variables (4) that vary significantly (*p* < 0.05), indicating that these pairs of horses have quite a different technique in performing piaffe. In general, the Lusitanos perform piaffe using a similar technique, as no more than one variable is significantly different between pairs of horses. Inter-horse and intra-horse variabilities are illustrated by heat maps in Figure 5 which show the mean values and CoVs for each variable on a horse-by-horse basis. The shades of blue (forelimbs) and red (hindlimbs) are on a gradient with larger magnitudes represented by darker shading.

**Figure 6:**
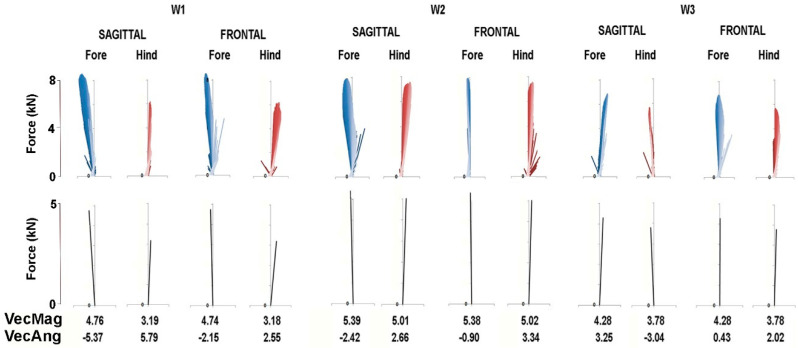
Force vector diagrams (upper panel) of Warmblood horses for the same stance phases as in Figure 3. In the sagittal plane vector diagrams, propulsive longitudinal forces are positive to the right. In the frontal plane vector diagrams, medially directed transverse forces are positive. Summary vectors derived from each vector diagram are shown in the lower panel together with the values for the mean vector magnitude (VecMag) and mean vector angle (VecAng).

**Figure 7:**
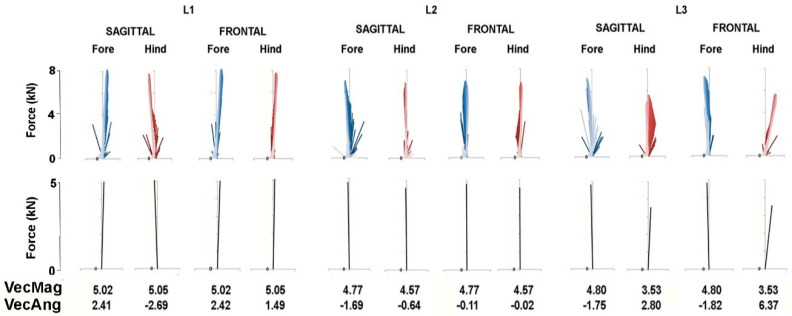
Force vector diagrams (upper panel) of Lusitano horses for the same stance phases as in Figure 4. In the sagittal plane vector diagrams, propulsive longitudinal forces are positive. In the frontal plane vector diagrams, medially directed transverse forces are positive. Summary vectors derived from each vector diagram are shown in the lower panel together with the values for the mean vector magnitude (VecMag) and mean vector angle (VecAng).

The force vector diagrams in Figure 6 and Figure 7 show the GRF vector throughout the stance phase in the sagittal and frontal planes together with values for VecMag and VecAng in the six horses for which data were available for both fore and hindlimbs. In general, the vectors in each diagram form a narrow envelope that is oriented close to the vertical. Comparing the fore and hindlimbs, VecMag is markedly higher in the forelimbs in W1 and L3, slightly higher in the forelimbs in W2 and W3 and almost equal in L1 and L2. Most of the horses follow a pattern of braking in the forelimbs and propulsion in the hindlimbs, but two horses—W3 and L1—have the opposite pattern, so the hindlimbs are braking and the forelimbs are producing propulsion. Note that VecAng is usually directed more medially in the hindlimbs compared with the forelimbs in the frontal plane views.

## 4. Discussion

This study is believed to be the first detailed report of the three-dimensional GRFs in the fore and hindlimbs of horses performing the piaffe. Analysis of the data presented here is a first step toward understanding the effects of the most highly collected gaits on the musculoskeletal tissues of high-level dressage horses.

The dressage rules of the Fédération Equestre Internationale (FEI) describe the characteristics and performance of the piaffe. The traits relevant to this study are paraphrased here in italics. *Piaffe is a highly collected, cadenced, elevated diagonal movement giving the impression of remaining in place. Each diagonal pair of legs is raised and returned to the ground alternately, with spring and an even cadence. Piaffe must always be animated by a lively impulsion and characterised by perfect balance. While giving the impression of remaining in place, there may be a visible inclination to advance*. Faults to be penalized by judges include: *moving too much forward, moving even slightly backwards, a lack of clear diagonal steps, crossing the legs or swinging sideways* [1]. In biomechanical terms, the horse must balance on a diagonal pair of limbs while raising the other diagonal pair in a controlled manner and briefly holding the swing phase limbs in their most elevated position. This implies that the horse must be stable while balancing on a diagonal pair of limbs.

In the sport of dressage, collection and self-carriage are highly prized characteristics that are dependent a good control of balance. In the diagonally coordinated gaits, there is a progressive decrease in speed and a progressive increase in the degree of collection from collected trot to passage to piaffe. Passage is a diagonally synchronized movement with two suspension phases per stride performed at slow speeds in the range of 1.2–1.9 m/s [3,4,5]. Like piaffe, it is performed only at the highest levels of dressage competition and might be regarded as an intermediate gait between trot and piaffe. Passage, with its slow forward velocity, relies on a combination of static and dynamic equilibrium. Horses control the pitching rotation of the body by adjusting limb placement, limb timing and GRF distribution between the fore- and hindlimbs [5]. Since piaffe is performed in place, it has a greater reliance on static equilibrium than passage with adjustment of GRFs likely to play a major role in maintaining balance.

As speed decreases, temporal kinematics change to facilitate the maintenance of balance. Increases in stance duration result in longer periods of overlap between grounded limbs, especially in the piaffe, which has no suspension phases [3,6]. An increase in the number of overlapping limb contacts enlarges the base of support which helps to maintain stability. Longer stance durations also allow more time to generate the necessary vertical impulse with lower peak GRFv. Table 3 compares the stance duration, peak GRFv and vertical impulse as reported here for the piaffe with corresponding values for the same horses performing collected trot and passage [8]. Stance durations are significantly longer and Peak GRFvert is significantly lower in the piaffe than in trot or passage in both fore and hindlimbs. Vertical impulse is higher in the piaffe than the collected trot in the hindlimbs only.

Perhaps the most notable feature of the GRFs in the piaffe is the variability, not only between horses, but also from step-to-step within individual horses. Due to the need to support body mass, variables related to the vertical GRF component are relatively consistent (CoV < 10%), whereas the longitudinal and transverse GRF variables have higher CoV (25–68%). Passage also has a large CoV for the longitudinal GRF variables, whereas the values in collected trot are around half those in piaffe [10].

One source of GRF variability in the piaffe is that, since it is an artificial gait, horses may learn to perform it with different techniques, for example, slight differences in limb placements relative to the body and its COM that affect GRF distribution between the limbs. The fact that piaffe is performed with little, if any, forward velocity implies a greater reliance on static equilibrium compared with the more forward-moving diagonal gaits. Raising a diagonal pair of limbs from the ground perturbs the horse’s balance and the grounded limbs react by applying forces in the horizontal plane to maintain equilibrium. Horses sense their body position relative to gravity and their surroundings through a combination of visual, vestibular, and proprioceptive inputs that provide feedback from the peripheral musculoskeletal system to the brain [11]. The core musculature stabilizes the trunk and neck segments while the extrinsic limb musculature positions these segments relative to the limbs [12]. The intrinsic limb musculature is responsible for the orientation and stabilization of the limb segments [13]. Insufficient strength in any of these muscle systems is likely to interfere with a horse’s ability to maintain balance in piaffe. Thus, high level dressage horses require great strength and finely tuned neuromotor control; this is acquired and further developed through a prolonged program of dressage training.

Force vector diagrams facilitate the visualization of the planar GRFs and are useful for comparison with published values for other gaits [10]. Unlike the trot and passage, in which the COM moves forward continuously and at fairly constant speed relative to the diagonal base of support [14], in piaffe the COM should not progress either forward or backward. When comparing the diagrams in Figure 6 and Figure 7 for piaffe with those of passage and collected trot [10], it is evident that the vector envelope becomes narrower and the VecAng approaches zero as speed decreases. The proximity of VecAng to zero in piaffe indicates that the mean force vector is aligned almost vertically, which is consistent with performing the piaffe in place. In the absence of forward motion, the limbs are lifted and lowered with little, if any, protraction and retraction [3]. The force vector becomes closely aligned with the COM velocity vector, which implies relatively large collisional energy losses in the process of reversing the movement of the COM from downward to upward during the stance phase [15]. This may contribute to the need for a large vertical impulse in piaffe.

The piaffe is required to be performed in place only at the highest levels of competition due to the greater difficulty of maintaining the diagonal coordination pattern as speed decreases. When a movement is performed with zero or constant velocity, it implies that the net longitudinal impulse per stride is zero. In other words, the resultant sagittal plane GRF vectors for the fore and hindlimbs should be equal in magnitude and opposite in direction. The summary force vectors in the lower panels of Figure 6 and Figure 7 and the values for VecMag and VecAng show how close the horses come to satisfying these conditions in most of the steps shown. However, this is not always the case; if the summed fore and hindlimb vectors differ sufficiently from zero, then the horse will take a small step forward or backward. Interestingly, in some steps, the hindlimb produces net braking and the forelimb produces net propulsion, which is contrary to their roles in forward locomotion. Future studies will explore relationships between limb kinematics, GRFs and body movements.

A limitation to the study is the small number of horses; it is not easy to recruit horses that are sufficiently highly trained and trainers who are willing to participate in the time-consuming data collections. A difficulty inherent in studying piaffe is that it is rare to capture a clean force plate contact, hence the need to filter out impact spikes in some of the GRF traces at the end of stance. Both the filtering process and the presence of GRF spiking from over-lapping limbs are likely to be associated with small inaccuracies in force values during the terminal parts of the stance.

## 5. Conclusions

Piaffe requires the horse to perform a difficult balancing feat in a specific posture. Knowledge of the associated GRFs will be helpful in understanding the effects on the musculoskeletal system and the unique stresses on the limbs in this artificial gait. The ability to generate an appropriate GRF profile affects the quality of the piaffe while the limb loading patterns influence susceptibility to injury. These will be investigated further by combining force data with kinematic analysis to evaluate the effects of GRFs on the performance of piaffe.

## Figures and Tables

**Table 1:** Temporal and ground reaction force variables for a group of horses (N = 7) performing the piaffe. Comparisons between the fore- vs. hindlimbs and between individual horses were made using ANOVA for normally- distributed variables (peakGRFv, time of peak GRFv, peakGRFpr, time of peak GRFpr, peakGRFmed, peak GRFlat) and the Mann–Whitney U test (fore vs. hind) and Kruskall–Wallis test (between horses) for all other non-normally distributed variables. Significant differences (*p* < 0.05) are bolded. CoV: coefficient of variation, n: number of stance phases. Vertical GRF (GRFv) is positive upwards; braking GRF (GRFbr), propulsive GRF (GRFpr), positive cranially; and medial GRF (GRFmed), lateral GRF (GRFlat), positive medially. Note: Fore vs. hind comparison was omitted from analysis for peak GRFlat, due to the lack of data on the hindlimb.

Variable	Fore				Hind				Fore vs. Hind (*p* Value)	Inter-Horse (*p* Value)
	n	Mean	SD	CoV (%)	n	Mean	SD	CoV (%)		
Stance duration (s)	28	0.6	0.1	17	24	0.65	0.2	31	0.963	**<0.001**
Peak GRFv (N/kg)	28	7.39	0.99	13	24	6.41	0.64	10	**<0.001**	**<0.001**
Time of peak GRFv (% stance)	28	54	9.3	17	24	49	10.1	21	0.180	**<0.001**
Vertical impulse (N.s/kg)	28	2.75	0.46	17	24	2.58	0.63	24	0.084	**<0.001**
Peak GRFbr (N/kg)	16	−0.50	0.34	68	16	−0.40	0.21	53	0.445	0.170
Time of peak GRFbr (% stance)	16	46	18.2	40	16	52	21.8	42	0.491	**0.009**
Peak GRFpr (N/kg)	19	0.37	0.15	41	13	0.50	0.26	52	0.679	**0.003**
Time of peak GRFpr (% stance)	19	67	23.1	34	13	57	22.8	40	0.480	**<0.001**
Peak GRFmed (N/kg)	16	0.32	0.17	53	23	0.40	0.18	45	0.174	**0.015**
Time of peak GRFmed (% stance)	16	68	17.1	25	23	60	21.8	36	0.301	0.053
Peak GRFlat (N/kg)	20	−0.29	0.10	34	1	−0.23	-	-	-	0.087
Time of peak GRFlat (% stance)	20	42	16.6	40	1	36	-	-	-	0.092

**Table 2:** Significant (*p* < 0.05) Bonferroni-adjusted, between horse comparisons of combined fore and hindlimb GRF variables (A–F). A: stance duration, B: peak GRFv, C: time of peak GRFv, D: vertical impulse, E: time of peak GRFbr, F: peak GRFmed. Post hoc testing not performed for peak GRFpr, time of peak GRFpr and peak GRFlat, due to insufficient data from at least one horse.

	W1	W2	W3	L1	L2	L3	L4
W1	-						
W2	A	-					
W3	B	AB	-				
L1	BC	BF	C	-			
L2	ADE	E	ABDE	B	-		
L3	AD	DEF	AD	C	B	-	
L4	ABCD	BDE	AD	F	B	F	-

**Table 3:** The comparison of stance duration, peak vertical force and vertical impulse in collected trot, passage and piaffe. Data for collected trot and passage are from [8]. MANOVA was used to test the difference between gaits with post hoc Bonferroni adjusted pairwise comparisons. Significant main effects (*p* = 0.001) were found between gaits. Post hoc significance (*p* < 0.05) is denoted by a: trot different from passage; b: trot different from piaffe; c: passage different from piaffe.

	Forelimbs			Hindlimbs		
	Trot	Passage	Piaffe	Trot	Passage	Piaffe
Stance duration (s)	0.38 ^ab^ (0.03)	0.47 ^bc^ (0.00)	0.60 ^ac^ (0.10)	0.36 ^b^ (0.03)	0.49 ^c^ (0.08)	0.65 ^bc^ (0.02)
Peak vertical GRF (N/kg)	10.06 ^b^ (0.54)	9.74 ^c^ (1.16)	7.39 ^bc^ (0.99)	8.28 ^b^ (0.79)	8.48 ^c^ (1.09)	6.41 ^bc^ (0.64)
Vertical impulse (N·s/kg)	2.44 ^a^ (0.20)	2.85 ^a^ (0.08)	2.75 (0.46)	1.81 ^ab^ (0.14)	2.43 ^a^ (1.03)	2.58 ^b^ (0.63)

## Data Availability

Data published in a supplementary file.
